# DEMETRA—A Seismic Noise Survey at the Maccalube di Aragona Mud Volcanoes (Southern Italy): Results and Perspectives

**DOI:** 10.3390/s25226975

**Published:** 2025-11-14

**Authors:** Simona Petrosino, Paolo Madonia, Daniele Gucciardo, Paola Cusano

**Affiliations:** 1 Istituto Nazionale di Geofisica e Vulcanologia (INGV), Sezione Osservatorio Vesuviano Via Diocleziano, 328, 80124 Naples, Italy; paola.cusano@ingv.it; 2 Istituto Nazionale di Geofisica e Vulcanologia (INGV), Sezione Osservatorio Etneo Piazza Roma, 2, 95125 Catania, Italy; paolo.madonia@ingv.it; 3 Legambiente Sicilia, Gestione Riserva Naturale “Maccalube di Aragona” Via Salvatore La Rosa, 53, 92021 Aragona, Italy; danielegucciardo@gmail.com

**Keywords:** seismic survey, mud volcanoes, HVSR, polarization, Maccalube di Aragona

## Abstract

On 22–23 April 2025, a seismic noise survey was conducted at the Maccalube di Aragona, a mud volcano field located in Sicily (southern Italy), with the aim of characterizing the background signal associated with vent activity and the shallow subsurface structure. The experiment, named DEMETRA (DEnse MaccalubE TRomino Acquisition), was carried out within the framework of the multidisciplinary INGV-PROMUD research project, which aims to identify key indicators of mud volcano activity and potential precursors of paroxysmal events. Ambient seismic noise was recorded at 21 sites using a three-component, 24-bit digital tromograph. Measurements were conducted with a dense spatial sampling scheme covering both vent areas and peripheral zones. Preliminary data analyses included spectral estimates, computation of horizontal-to-vertical spectral ratio (HVSR) curves and evaluation of the polarization patterns. The HVSR curves do not display clear amplification peaks but rather show deamplification at specific sites. The polarization patterns exhibit spatial consistency across the vent areas. In addition, transient signals were identified in the background noise at some sites; based on their spectral and polarization characteristics, these signals are possibly associated with degassing, mud emissions, or bubbling phenomena. The dense spatial coverage of the DEMETRA experiment provides a valuable dataset for investigating subsurface properties and dynamic processes in an active mud volcano environment.

## 1. Introduction

Mud volcanoes (or sedimentary volcanoes) are surface manifestations of deep-seated overpressured fluids, mainly composed of water and gas (mostly methane and carbon dioxide), mixed with argillaceous material and fine sediments. These fluids migrate upward through preferential pathways such as faults or lithological discontinuities, producing outflows of semi-liquid mud at the surface. Mud volcanism commonly occurs in tectonically active compressional settings or in sedimentary basins undergoing rapid burial, where high pore-fluid pressures can exceed lithostatic stress and trigger eruptive activity [1,2,3,4]. The morphology and size of these structures depend on factors such as eruption frequency, fluid viscosity and overpressure intensity and may range from small domes to extensive fields several square kilometers wide. Mud volcanoes are typically in a quiescent stage, and their eruptions are of short duration (from hours to a few days). Violent eruptions, known as “overturning”, occur with sudden bursts of mud breccia reaching heights of several tens of meters, accompanied by gas flow and water seepage.

In Italy, mud volcanism is well documented across the Apennine and Sicilian thrust belts, with active onshore and offshore occurrences [1,5,6], and at least 87 structures have been identified. Italian mud volcanoes show a variety of sizes and shapes, with about 20% covering a surface area larger than 500 m^2^, and only 5% exceed 2 m in height [5].

Among the Italian onshore mud volcanoes, the Maccalube di Aragona in southern Sicily constitute one of the most remarkable and active fields, known since historical times. Its persistent activity, lasting for at least 2500 years and marked by both continuous low-energy degassing and episodic paroxysmal eruptions, makes it an ideal natural laboratory to directly observe geochemical, geophysical, and geomorphological processes driving mud volcanism. The name “Maccalube” derives from the Arabic word maqlūbah, meaning “overturning,” referring to the sudden eruptions that can produce significant surface changes. The field, located near the town of Aragona (Agrigento), covers an area of about 1.4 km^2^ and consists of several small cones (from few centimeters to half a meter in height) emitting mud, water, and gas, mostly CH_4_ and a small percentage of CO_2_ [6,7]. In the northern part of the Maccalube area, there are also two main pools (about 3 m in diameter) with bubbling high-pH (8.7) water and gas, whose composition is similar to that of the main vents [8]. The activity of the main emission vents is typically weak and continuous, but occasionally interrupted by violent paroxysmal events, as occurred in 2008, 2014, and, most recently, in 2020 [9]. The 2014 paroxysm occurred on 27 September and was preceded in August by the opening of large-scale ground fractures. The emission vents were located nearby a NW–SE structural lineament Gattuso et al. [9]. The eruption involved mud mixed with water and gas, reaching a column height of 15 m and covering a surface area of 7525 m^2^. Two children tragically died, buried by the erupted mud deposits. On 19 May 2020, a new paroxysmal event occurred in the southeastern part of the main vent area, emitting a mud volume of 18,196 m^3^ and covering an area of 8721 m^2^, with a maximum deposit thickness of 3.7 m. This 2020 paroxysm occurred in a medium–high density area of emission points, where a NE–SW structural lineament has been highlighted by Gattuso et al. [9]. Since 2023, the emission vents have further migrated toward the southeast. A new major overturning occurred on 30 August 2025, while this study was being completed. The event is currently under detailed multidisciplinary investigation.

Recent studies have shown that seismic noise analysis, particularly through the Horizontal-to-Vertical Spectral Ratio (HVSR) technique [10], can provide valuable insights into the subsurface structure of fluid-saturated systems, including both hydrocarbon reservoirs and mud volcanoes. HVSR measurements in hydrocarbon fields often reveal spectral minima in the 1–6 Hz range [11,12], interpreted either as resonance effects within the fluid-filled reservoir [13] or as poro-elastic responses of the medium [14]. Similar features have been observed in mud volcano areas, such as Salse di Nirano (northern Italy), Santa Barbara, and Salinelle (both in southern Italy), though with HVSR minima often occurring below 1 Hz [15,16,17], and in the 1–10 Hz frequency band [16,18]. This deamplification effect has been attributed to the presence of a velocity contrast between a fluid-rich reservoir (containing mainly mud, water, and methane) and the surrounding rock matrix [15,18]. These studies suggest that HVSR curves can be used to infer and characterize the presence, depth, and properties of fluid reservoirs in mud volcano systems.

In parallel, polarization analysis [19] of seismic noise has emerged as a valuable tool to assess the directional properties of the wavefield and identify dominant sources [16]. Moreover, the spatial pattern of polarization parameters can reveal structural heterogeneities or anisotropy, as ground motion often occurs orthogonally to the azimuth of the main fracture field [16,20,21]. Conversely, polarization parallel to the fault strike has been observed as a consequence of trapped waves in low-velocity damaged rocks with high crack density [22], or wave propagation in fluid-saturated fractures [21].

Additionally, time-domain analysis of transient seismic events—such as short-duration, high-amplitude bursts—has emerged as a complementary approach for investigating fluid movement and episodic degassing phenomena. For example, at Salse di Nirano, various types of high-frequency signals (up to 40 Hz) have been identified [17,18]. Among these, drumbeats exhibit long durations (on the order of several seconds) and irregular timing, while drumrolls consist of very regular sequences of identical, short (<1 s), monochromatic pulses. Such signals are likely driven by local pressure variations within the plumbing system, inducing the migration of bubbling fluids toward the surface [17,18]. Altogether, seismic noise measurements are proving to be a powerful tool for delineating shallow subsurface structures, detecting buried gas/fluid pockets, and understanding the dynamic behavior of mud volcano systems [15,18,23,24,25].

In this study, we present the results of the first dense seismic noise survey conducted in April 2025 at the Maccalube di Aragona. The experiment, called DEMETRA (DEnse MaccalubE TRomino Acquisition), aimed at (i) characterizing the noise wavefield through spectral, HVSR, and polarization analyses; (ii) identifying transient phenomena potentially related to fluid movements; and (iii) assessing the spatial variability of recorded signals in relation to shallow subsurface structure and gas emission zones. This approach represents the first systematic seismic noise investigation ever performed at the Maccalube di Aragona mud volcano field. The DEMETRA data will be integrated with those collected during continuous seismic noise acquisition within the framework of the INGV–PROMUD research project (https://progetti.ingv.it/it/promud, accessed on 10 November 2025), a multidisciplinary three-year program (2023–2025) whose final goal is to define monitoring protocols for mud volcanoes through assessment of their background activity and identification of potential precursors of paroxysmal events.

## 2. Materials and Methods: The DEMETRA Survey

**Figure 1:**
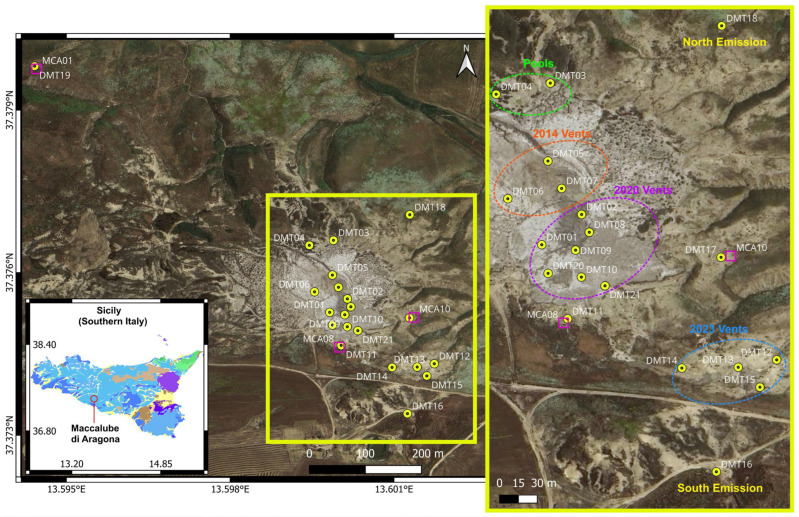
Satellite view of the Maccalube di Aragona mud volcano field and locations of the measurement sites (yellow circles). The three magenta squares indicate the permanent seismic stations deployed for the PROMUD project. The 2014, 2020 and 2022 vent areas are outlined by orange, violet and blue ellipses. The pool zone is indicated by the green ellipse. The northern and southern emission sites are labelled in yellow. The position of the study area is marked by the red circle on the lithological map of Sicily. For a full description of the lithotypes refer to the EGDI 1:1 Million pan-European Surface Geology map (https://maps.europe-geology.eu/, accessed on 30 October 2025). Base map: Google Earth, Image©2025 Airbus.

The DEMETRA (DEnse MaccalubE TRomino Acquisition) survey was conducted on 22–23 April 2025 at the Maccalube di Aragona mud volcano field. The instrumental set up was designed to record seismic noise with dense spatial sampling, covering both the active vent zones and adjacent areas. A total of 21 measurement sites were selected to ensure spatial representativeness of both active and peripheral zones, and to capture the variability of structural/geological and fluid-dynamic conditions across the field (Figure 1). These include the following areas:

- the 2014 vents;
- the 2020 vents;
- the 2023 vents;
- the northern pools;
- two isolated emission sites located outside the main field (one to the north and one to the south);
- three points co-located with the permanent seismic stations MCA08, MCA10, MCA01 [26] from the PROMUD project, the latter positioned approximately 800 m from the main field.

Overall, the survey area spans approximately 30,000 m^2^, allowing for a detailed sampling of both active and quiescent sectors (Figure 1, Table 1). All recordings were performed using a three-component, 24-bit digital tromograph (Tromino™ ZERO, manufactured by Moho s.r.l., Marghera (Venice), Italy, https://moho.world/, accessed on 10 November 2025), which integrates high-sensitivity velocimetric sensors. The instrument was placed directly on the ground surface, carefully leveled and oriented to magnetic north. Each measurement lasted 30 min, with a sampling rate of 128 Hz and sensitivity set to ±0.5 mm/s (high gain mode). Data were stored internally on SD memory card and downloaded using the Grilla Rel. 9.9.0 software package (https://www.tromino.it/frameset-grilla.htm, accessed on 31 January 2025) [27].

During the campaign, each site was documented through detailed photographs and GPS positioning. Coordinates and elevations were determined using GPS data with an estimated horizontal accuracy of approximately 4 m. Table 1 reports the full list of sites, their coordinates (WGS84), elevations, and recording times.

**Figure 2:**
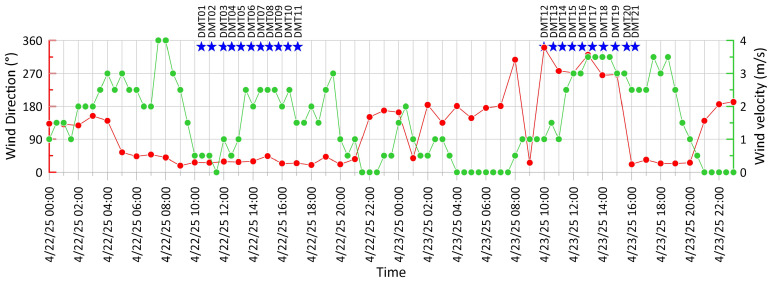
Wind velocity and direction on 22–23 April 2025.

Environmental conditions during the campaign were generally favorable: the sky was clear on 22 April and during the morning of 23 April. The early afternoon of 23 April was partly cloudy, with light rainfall affecting only the final two recordings. Wind velocity ranged from 1.5 to 3 m/s, with a dominant NNE direction on the first day, and from 2 to 4 m/s on the second day, turning from W–WNW in the morning to NNE in the early afternoon, when it reached its highest intensity (Figure 2). Meteorological data were retrieved from a local weather station [26] and from the SIAS database (http://www.sias.regione.sicilia.it/corpo_dati.htm, accessed on 31 May 2025).

## 3. Results

### 3.1. Spectral Analysis

**Figure 3:**
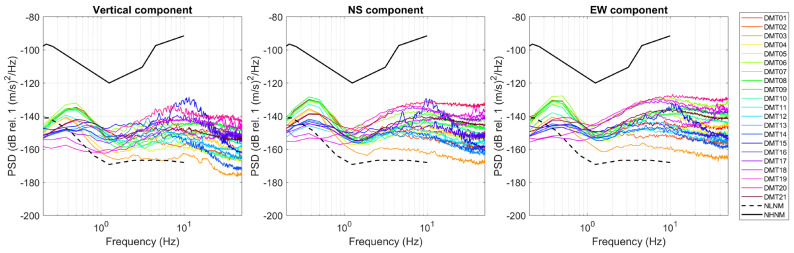
Power Spectral Density of the seismic signals recorded at the sampled sites. The dashed and continuous black curves represent the New Low Noise Model (NLNM) and New High Noise Model (NHNM) [28].

Spectral analysis of the seismic recordings was performed to estimate both the frequency content and the overall level of noise during the survey period. For all the sampled sites, the Power Spectral Density (PSD) of the three motion components was computed as the average over 30 min time windows; their comparison reveals a dominant spectral peak below 1 Hz (between 0.3 and 0.9 Hz), whose amplitude varies significantly across the sites (Figure 3). This low-frequency peak is generally more pronounced at points located in the vicinity of the 2014 and 2020 vent areas, suggesting a potential link with persistent subsurface processes in those zones. In addition, several sites—namely DMT06, DMT07, DMT08, DMT09, DMT10 (sampled in the afternoon of 22 April), and DMT17, DMT19, DMT20, DMT21 (sampled in the afternoon of 23 April)—also exhibit a broader spectral contribution between 2 and 30–40 Hz, especially on the EW components. In some cases (e.g., DMT17, DMT19, DMT20, all located outside the vent area), the high-frequency spectral amplitude exceeds that of the low-frequency peak. The persistence of the aforementioned spectral components over the entire recording duration (30 min) is confirmed by the spectrograms. Additionally, some stations exhibit narrow-band, high-frequency peaks associated with short-duration transient signals. These transients occur sporadically and will be discussed in more detail in the dedicated Section 3.4.

The calculated PSD curves fall within the range defined by the New High Noise Model (NHNM) and New Low Noise Model (NLNM) of Peterson [28], and are generally closer to the NLNM curve, indicating overall low ambient noise levels at all the sites. At low frequencies (<0.5 Hz), the PSD values slightly fall below the NLNM, suggesting particularly quiet conditions in that range. This behavior confirms that the recorded seismic noise does not show anomalous deviations from the reference models.

### 3.2. HVSR Estimates

Spectral ratios between the horizontal and vertical components were computed to identify potential resonance frequencies, providing preliminary insight into the mechanical properties of the shallow subsurface. Indeed, the Horizontal-to-Vertical Spectral Ratio (HVSR) technique [10] is particularly well suited to identify impedance contrasts at the transition between rock types with different physical–mechanical characteristics. The analysis was carried out using Grilla Rel. 9.9.0 software package (https://www.tromino.it/frameset-grilla.htm, accessed on 31 January 2025) [27]. Each 30 min three-component seismic recording was segmented into non-overlapping 30 s time windows, a duration that allows the resolution of HVSR frequency peaks greater than 0.33 Hz. An STA/LTA-based anti-trigger algorithm was used to exclude transient disturbances, retaining only the most stationary windows. In our dataset, due to the relatively low level of disturbances, a large proportion of each signal, ranging between 92% and 98% depending on the site (Table A1, Appendix A), was deemed suitable for HVSR analysis. Each windowed segment was detrended, and its Fourier amplitude spectrum computed for each channel. Spectra were smoothed using a triangular window with a bandwidth equal to 10% of the central frequency. The Konno–Ohmachi smoothing function (with a filter coefficient b = 40) was also tested and produced comparable results. The HVSR was then calculated as the geometric mean of the two horizontal components divided by the vertical one:

$$HVSR\left(f\right)=\;\frac{\sqrt{NS(f)\cdot EW(f)}}{V(f)}$$

**Figure 4:**
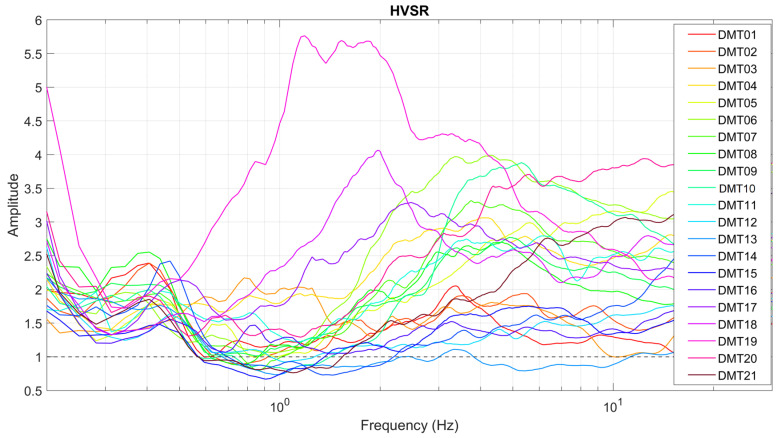
HVSR curves for all the sampled sites.

The final HVSR curve, with the 95% confidence interval relative to its amplitude, was obtained by averaging the HVSR computed for each individual time window. The HVSR curves for all the 21 sites are shown in Figure 4.

To assess the robustness of the obtained HVSR curves, we applied the SESAME [29] criteria, which are divided into two groups (Appendix A):

- Reliable curve criteria (3 conditions)—These relate to the minimum required length of the signal as a function of the frequency of the peak. All three criteria must be satisfied for a reliable result. If not, the analysis should typically be repeated using a different window length.
- Clear peak criteria (6 conditions)—These assess the statistical significance and clarity of the HVSR peak. At least 5 out of 6 should be met to classify the peak as clear. Failing to meet this threshold does not invalidate the result but rather categorizes the peak as unclear. Typical examples include low frequency (*f* < 1 Hz) peaks, broad peaks and multiple peaks. In these cases, according with SESAME [29] guidelines, further inspection is recommended (for example, by varying the smoothing bandwidth, examining HVSR curves from individual windows, checking the low frequency asymptote, verifying whether a peak appears within the standard deviation interval), in order to determine whether the HVSR pattern reflects a limitation of the analytical approach or a genuine site characteristic.

In our case, all 21 HVSR curves satisfy the reliability criteria (Table A1, Appendix A), confirming the validity of the data processing workflow. In fact, for 30 min recordings, a 30 s segmentation yields a sufficient number of time windows (typically ≥ 20) to ensure statistical robustness [29,30]. However, none of the sites fully met the threshold of satisfying 5 out of 6 clarity criteria for the HVSR curves, except for DMT01, which technically scored 5 out of 6. Yet, the third criterion, the minimum amplitude threshold, was only barely met, just above the required level, meaning that the effective score in terms of satisfied criteria would be closer to 4 out of 5. As such, all the observed peaks, which appear as flat or broad features in the 1–10 Hz range, should be considered as unclear. Since we carefully verified that the results were independent of the choice of the parameters used for the analysis, the absence of a sharp peak does not seem to result from methodological limitations.

Interestingly, several sites show an HVSR minimum (values < 1), which may indicate a phenomenon of deamplification. This feature is observed at sites DMT02, DMT07, DMT08, DMT10, and DMT21, primarily located in the 2020 vent area, with troughs centered between 0.6 and 1.5 Hz. Even more pronounced minima, spanning a broader and higher frequency range (0.6–2 Hz and extending up to ~10 Hz), are detected at DMT12, DMT13, DMT14, and DMT15, which are located in the 2023 vent area.

### 3.3. Polarization Analysis

The polarization parameters of the ambient noise wavefield were extracted using the covariance matrix method [19]. Diagonalization of the covariance matrix of the three-component signals allowed us to compute the following parameters:

1. Rectilinearity (RL), ranging from 0 for fully spherical motion to 1 for purely rectilinear motion;
2. Azimuth of the polarization vector, defined as the clockwise angle between its horizontal projection and the geographic North;
3. Incidence angle, measured between the polarization vector and the vertical axis. An angle of 90° corresponds to horizontal propagation, whereas 0° indicates vertical incidence.

Polarization parameters were estimated using ad hoc developed Matlab scripts (available at: https://doi.org/10.17605/OSF.IO/KQTBP, accessed on 10 November 2025) [31]. The analysis was carried out in two frequency bands (0.2–1 Hz and 1–5 Hz). The time series were processed using sliding windows with a length equal to two cycles of the maximum period in each band, with a 50% overlap. Polarization parameters were then averaged over time to obtain stable estimates. The azimuthal spread of polarization directions was quantified using the resultant vector length R [32], a statistical measure of the concentration of azimuths around the mean direction. R ranges from 0 (maximum dispersion) to 1 (perfect alignment). An empirical threshold of R = 0.4 was adopted to distinguish between well- and poorly collimated azimuthal distributions [21,33].

**Figure 5:**
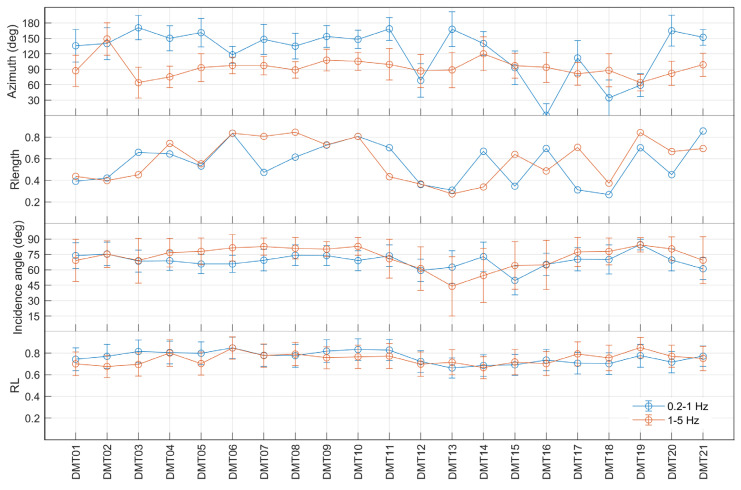
Mean values and standard deviations of the polarization parameters (azimuth, resultant length Rlength, incidence angle and rectilinearity RL) estimated for all the sampled sites in the 0.2–1 Hz (blue line) and 1–5 Hz (red line) frequency bands.

At all sites, rectilinearity values are ≥0.65 in both frequency bands, indicating the dominance of body and/or Love waves (Figure 5). Most sites exhibit incidence angles between 60° and 85° (Figure 5). In the 2014 and 2020 vent areas, slightly lower incidence angles were observed in the 0.2–1 Hz band compared to the 1–5 Hz band. In the 2023 vent area, the sampled sites show lower incidence angles (~40–60°) in the 0.2–1 Hz band (DMT012, DMT015) and in the 1–5 Hz band (DMT13, DMT14). Nevertheless, the overall values of incidence angles (>45°) suggest a predominantly horizontal polarization of wavefield in the analyzed frequency ranges.

The azimuthal distributions of the polarization vectors are generally well-collimated, with R values exceeding 0.4 at most sites (Figure 5). The 2014 and 2020 vent areas exhibit the highest R values, indicating strong directional consistency. In contrast, lower R values, and thus more dispersed azimuths, characterize the 2023 vent area, particularly in the 1–5 Hz band. Seismic noise recorded at sites DMT17 and DMT18 is also poorly polarized in the 0.2–1 Hz band.

**Figure 6:**
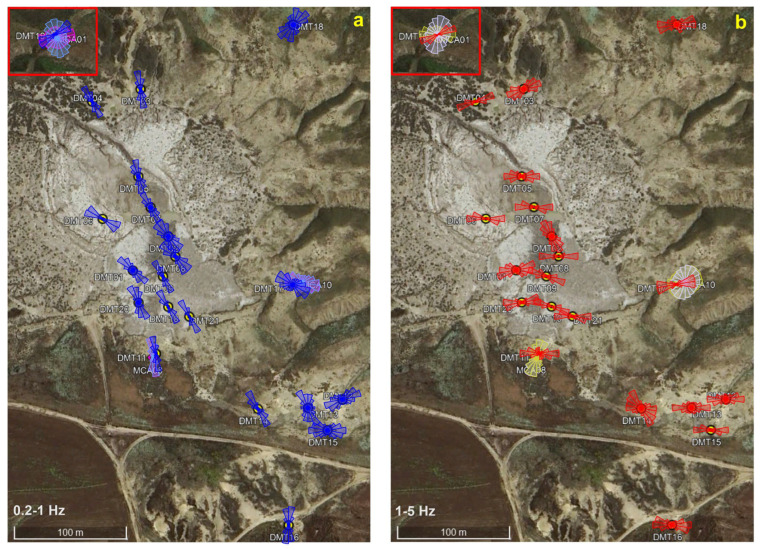
(**a**) Rose plots (in blue) of the polarization azimuth in the 0.2–1 Hz frequency band at the sites sampled during DEMETRA survey. DMT11, DMT17 and DMT19 distributions are compared with daytime (magenta rose plots) and night-time polarization (azure rose plots) at MCA08, MCA10 and MCA01, the permanent stations installed for the PROMUD project. (**b**) Rose plots (in red) of the polarization azimuth in the 1–5 Hz frequency band at the sites sampled during DEMETRA survey. DMT11, DMT17 and DMT19 distributions are compared with daytime (yellow rose plots) and night-time polarization (light violet rose plots) at MCA08, MCA10 and MCA01. Base map: Google Earth, Image©2025 Airbus.

To provide a comprehensive overview of the polarization pattern, rose diagrams were plotted on a map, offering both a spatial and statistical representation of azimuthal directions. In the 0.2–1 Hz band, well-collimated azimuths are observed in the 2014–2020 vent area and in the pool zone, with a dominant NNW–SSE direction (Figure 6, left panel). Conversely, the 2023 vent area (except site DMT14) exhibits greater azimuthal dispersion and no prevailing direction. In the 1–5 Hz band, a common E–W orientation is observed across all three vent areas, with the pool area showing a slightly ESW–WNE trend (Figure 6, right panel).

### 3.4. Identification of Transient Signals

A visual inspection of the waveforms and spectrograms was conducted to identify transient signals within the recorded noise, which may originate from gas or mud bubbling, or shallow fracturing. In several sites located: (a) in the 2020 and 2023 vent areas, (b) in proximity to the western pool, and (c) near the northern emission point, we identified short-duration, high-frequency transients superimposed on the background noise.

**Figure 7:**
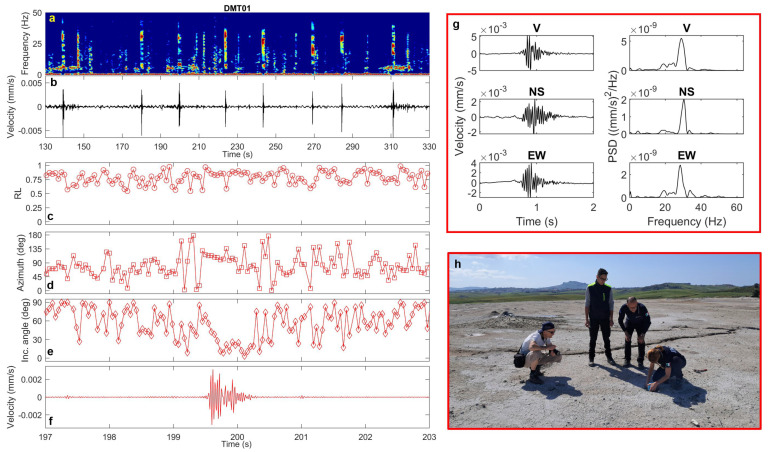
(**a**) Spectrogram, (**b**) vertical-component unfiltered seismogram recorded at DMT01 site (25 min-long seismic recording and spectrogram are also shown in the graphical abstract), (**c**) rectilinearity, (**d**) azimuth, (**e**) incidence angle, (**f**) zoomed vertical-component seismogram filtered in the 20–40 Hz frequency band, (**g**) three-component unfiltered waveforms (vertical, North–South and East–West) of a selected transient signal and the corresponding PSDs. (**h**) Installation of the Tromino at DMT01 site (photo courtesy of Daniele Gucciardo).

**Figure 8:**
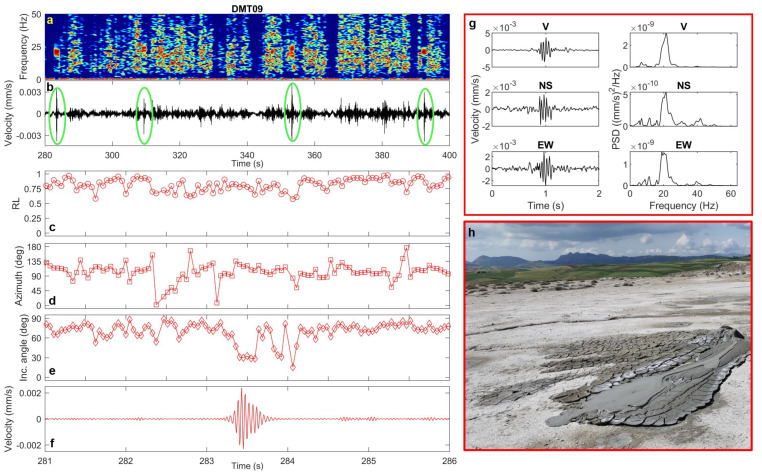
(**a**) Spectrogram, (**b**) vertical-component unfiltered seismogram recorded at DMT09 site (green ellipses mark the transient signals), (**c**) rectilinearity, (**d**) azimuth, (**e**) incidence angle, (**f**) zoomed vertical-component seismogram filtered in the 20–30 Hz frequency band, (**g**) three-component unfiltered waveforms (vertical, North–South and East–West) of a selected transient signal and the corresponding PSDs. (**h**) Mud volcano near DMT09 site; the same cone is also shown in the right panel of the graphical abstract(photo courtesy of Pierpaolo Pappacena).

**Figure 9:**
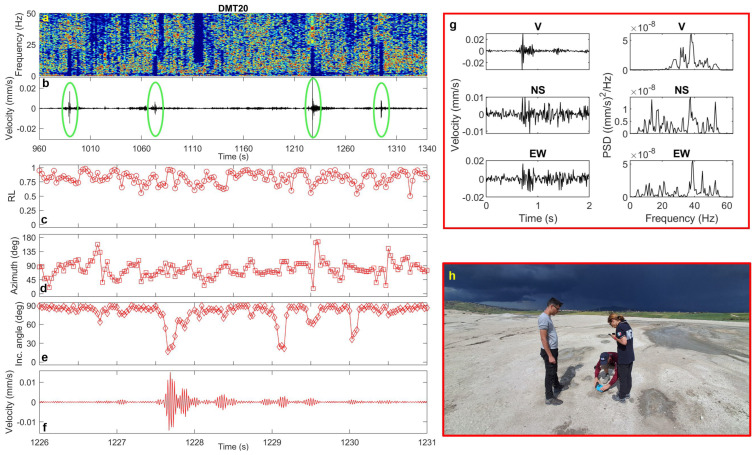
(**a**) Spectrogram, (**b**) vertical-component unfiltered seismogram recorded at DMT20 site (green ellipses mark the transient signals), (**c**) rectilinearity, (**d**) azimuth, (**e**) incidence angle, (**f**) zoomed vertical-component seismogram filtered in the 30–40 Hz frequency band, (**g**) three-component unfiltered waveforms (vertical, North–South and East–West) of a selected transient signal and the corresponding PSDs. (**h**) Installation of the Tromino at DMT20 site (photo courtesy of Daniele Gucciardo). A zoomed picture of one of the most active vents near this site is shown in the lower central panel of the graphical abstract (photo courtesy of Pierpaolo Pappacena).

In the 2020 vent area, these signals appear in the recordings at DMT01 (where they occur almost regularly every 20–40 s), as well as at DMT09 and DMT20. They are extremely short in duration (<1 s) and exhibit frequency content ranging from 20 to 40 Hz (Figure 7, Figure 8 and Figure 9). Polarization analysis of these pulses reveals high rectilinearity values and low incidence angles (20–30°), indicating predominantly sub-vertical polarization (Figure 7, Figure 8 and Figure 9). The azimuthal directions are consistent with the alignment of two of the most active cones, which continuously emit dense mud across the area.

**Figure 10:**
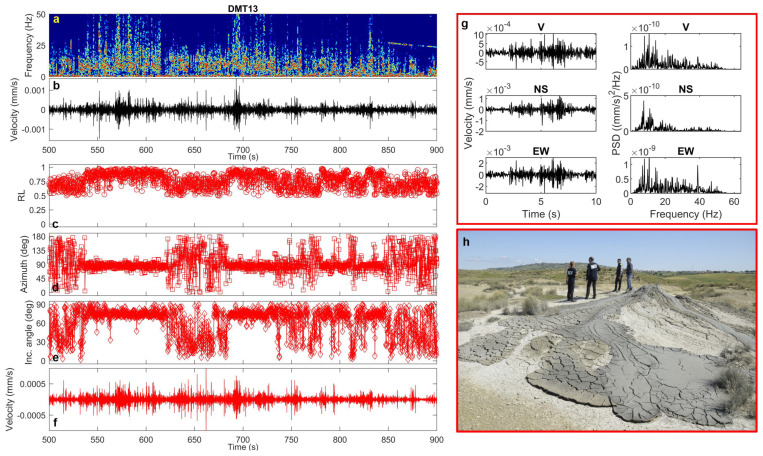
(**a**) Spectrogram, (**b**) vertical-component unfiltered seismogram recorded at DMT13 site, (**c**) rectilinearity, (**d**) azimuth, (**e**) incidence angle, (**f**) vertical-component seismogram filtered in the 5–20 Hz frequency band, (**g**) time-window of 10 s containing the three-component unfiltered waveforms (vertical, North–South and East–West) and the corresponding PSDs. (**h**) Mud volcano and field operation near DMT13 site (photo courtesy of Pierpaolo Pappacena).

In contrast, transients recorded at DMT13, near the most active mud cone in the 2023 vent area, tend to merge into continuous signals lasting several seconds, with dominant frequencies between 5 and 20 Hz (Figure 10). These signals are clearly recognizable through variations in the polarization parameters over time (Figure 10), which, compared to the background, exhibit higher rectilinearity, shallow incidence angles (~80°, indicating horizontal polarization), and well-collimated azimuths (~90°) compatible with the source-to-station direction.

**Figure 11:**
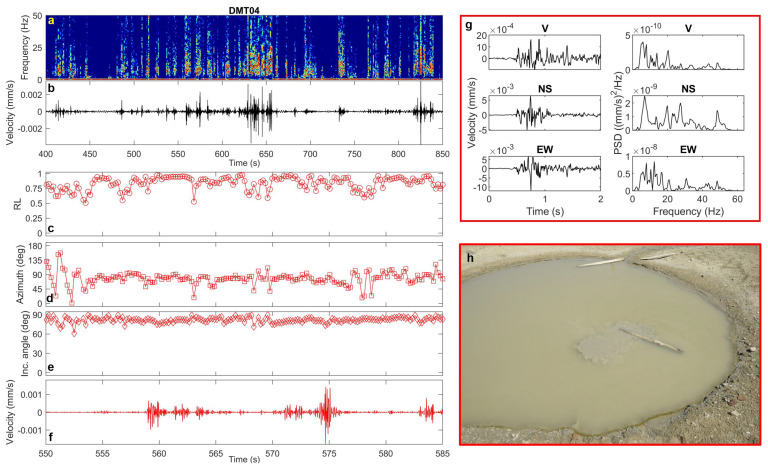
(**a**) Spectrogram, (**b**) vertical-component unfiltered seismogram recorded at DMT04 site, (**c**) rectilinearity, (**d**) azimuth, (**e**) incidence angle, (**f**) zoomed vertical-component seismogram filtered in the 5–20 Hz frequency band, (**g**) three-component unfiltered waveforms (vertical, North–South and East–West) of a selected transient signal and the corresponding PSDs. (**h**) Western pool near DMT04 site (photo courtesy of Pierpaolo Pappacena).

In the area north of the main vent field, which hosts the two pools, we sampled two sites. At DMT04, near the western pool, the transients (lasting 1–2 s) show frequency content between 5 and 20 Hz, high rectilinearity, and high incidence angles (~80°), suggesting horizontal polarization and a likely surface wavefield (Figure 11). The azimuths are again compatible with the location of the western pool, which showed active bubbling during the survey. Interestingly, no transient signals were identified at DMT03, near the eastern pool. This observation is consistent with concurrent geochemical evidence indicating that the eastern pool contains denser mud and lower gas content, whereas the western pool is richer in gas, resulting in visible bubbling phenomena.

**Figure 12:**
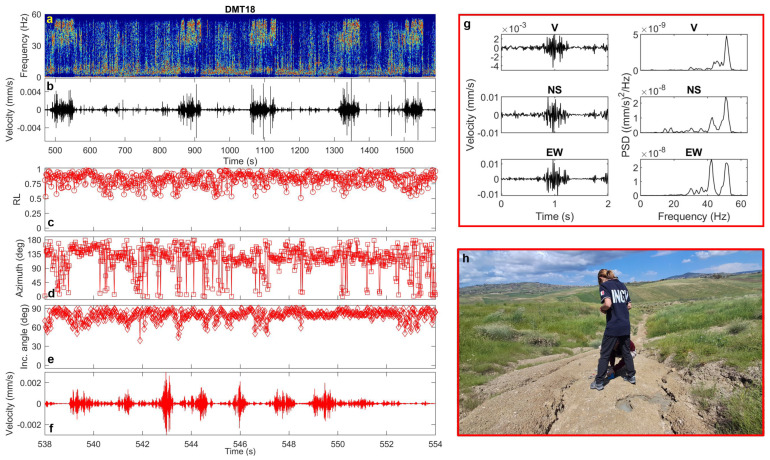
(**a**) Spectrogram, (**b**) vertical-component unfiltered seismogram recorded at DMT18 site, (**c**) rectilinearity, (**d**) azimuth, (**e**) incidence angle, (**f**) zoomed vertical-component seismogram filtered in the 40–60 Hz frequency band, (**g**) three-component unfiltered waveforms (vertical, North–South and East–West) of a selected transient signal and the corresponding PSDs. (**h**) Installation of the Tromino at DMT18 site, near the northern emission point (photo courtesy of Pierpaolo Pappacena).

Finally, at the northern emission point (DMT18), characterized by a small but continuous low-density fluid outflow, nearly periodic transients (approximately every 100 s) were observed. These consist of short pulses (1–2 s) that merge into ~1 min-long signal trains (Figure 12). The signals are characterized by extremely high-frequency content (40–60 Hz), variable incidence angles between 60° and 80°, and azimuths (Figure 12) consistent with the emission source.

## 4. Discussion

### 4.1. HVSR

The preliminary analyses carried out on the DEMETRA dataset offer an initial framework for interpreting seismic signals recorded at the Maccalube mud volcano field, in light of both subsurface structural properties and fluid-driven processes. The lack of clear peaks in the HVSR curves suggests that no substantial amplification is caused by the shallow subsoil structure. This behavior is consistent with the presence of fluid-saturated clay deposits, which can exert a strong damping effect on seismic wave propagation. In fact, the degree of saturation and the viscosity can significantly influence ground motion amplification. The seismic wave attenuation in fluid-saturated soils is generally greater than in dry conditions and also increases in materials with higher viscosity [34].

On the other hand, the observed deamplification at several sites provides valuable information. This pattern may reflect the presence of fluid-rich muddy material, which leads to a reduction in shear wave velocity [15,16], as well as strong seismic impedance contrasts between the fluid reservoir and the surrounding lithotypes at depth [18]. Notably, both the shape and frequency range of the HVSR minima show slight variations when moving from the 2020 vents southeastwards to the 2023 emission centers, suggesting spatial heterogeneities in the subsurface properties. This observation is consistent with the findings at the Salse di Nirano mud volcano by Brindisi et al. [18], who also reported spatial variations in HVSR minima and inferred the probable location of gas/fluid reservoirs based on their presence or absence. In their study, all measurements showing HVSR minima are concentrated in the areas corresponding to active emission vents.

While our interpretation of the HVSR curves at Maccalube di Aragona is plausible, further investigation is required to fully assess whether they result from genuine site effects related to subsoil conditions or are somewhat influenced by external factors such as wind intensity. As shown in Section 3.1 (Figure 3), some noise spectra exhibit a variable broad high-frequency content (2–40 Hz), which could be partially attributed to meteorological conditions. For instance, a significant increase in wind speed occurred on 22 April after 1:00 p.m. and during the late morning of 23 April (Figure 2), potentially enhancing high-frequency spectral amplitudes at more exposed sites. Although microseism related to environmental conditions is mostly confined to frequencies below 1 Hz [35], several studies have shown that wind coupling with the ground and/or local surface turbulence can generate shear stresses that primarily affect the horizontal components (especially of unburied instruments) even at frequencies up to 60–100 Hz [36,37,38,39]. Therefore, the influence of wind-related noise on our results cannot be completely ruled out [36,37,38,39], although low wind velocities (<5 m/s), such those measured during the survey (Figure 2), are generally not expected to drastically alter the HVSR curves [29].

### 4.2. Polarization

Polarization analysis reveals a wavefield predominantly polarized in the horizontal plane at most sites and directionality patterns that appear to align with the morphology and vent activity. In particular, at the 2014 and 2020 vent areas, the NNW–SSE orientation of the polarization vector in the 0.2–1 Hz band (Figure 6, left panel) is consistent with that of the two main structural lineaments identified by Gattuso et al. [9]. Specifically, the polarization direction, which is approximately orthogonal to the NE–SW lineament and parallel to the NW–SE one, can therefore be interpreted in two ways. It may reflect subsurface structural heterogeneities due to the decrease in stiffness of the medium fractures in the first case (orthogonal relationship, [15,20,21]), or alternatively, it may be associated with trapped waves in low-velocity damaged rocks or with wave propagation through fluid-saturated fractures in the latter case (parallel relationship) [21,22]. Conversely, the 2023 vent area (except site DMT14) exhibits greater azimuthal dispersion and no prevailing direction, likely reflecting weaker influence of the main structural discontinuities due to its greater distance from them.

In the 1–5 Hz frequency range the polarization azimuth shows a well-collimated E–W orientation across all three vent areas, without any apparent relation with the structural features (Figure 6, right panel). Comparing data from the same sites, the narrow azimuthal distribution at DMT11 and DMT17contrasts with the broader one retrieved at MCA08 and MCA10, the two permanent PROMUD stations [22]. Moreover, a difference of 20–25° in the average polarization incident angle [26] is observed, with the DEMETRA data showing higher values (nearly horizontal polarization) compared to those of the buried PROMUD stations. This means that the wavefield recorded by the Tromino instrument, placed directly on the ground, is dominated by waves propagating in the uppermost surface. The source of the near-surface wavefield noise could be related to shallow, small-scale soil heterogeneities, but we cannot exclude ground-wind coupled turbulences (as previously discussed for the spectral amplitudes), given that the observed E–W polarization is approximately orthogonal to the prevailing direction of the wind blowing during the survey (Figure 2).

Additional validation of the polarization results in the 1–5 Hz frequency band is therefore required to better assess possible directional effects due to meteorological factors, particularly wind direction and intensity. Conversely, such effects seem to be excluded in the 0.2–1 Hz band. At these lower frequencies, the noise is more sensitive to deeper structures, and thus less affected by near-surface anomalies. In fact, at sites DMT11 and DMT17, the polarization patterns are similar to those observed at the two permanent PROMUD stations MCA08 and MCA10 [26]. Importantly, the polarization azimuths at these permanent stations, recording continuously since July 2024, remain stable over time, regardless of weather changes. This stability supports the interpretation that the observed low-frequency directionality is governed by structural features rather than variation in the level of the microseismic noise (<1 Hz) [35].

Finally, a specific discussion is needed for the marked discrepancy observed at the peripheral site located ~800 m from the main vent area. In both frequency bands, the DMT19 measurement shows a well-collimated ENE–WSW polarization, in contrast with the nearly isotropic distribution observed at the nearby permanent station MCA01 (Figure 6). This behavior can be explained in terms of temporal variations: DMT19 captured a 30 min snapshot during which the dominant noise polarization is actually ENE–WSW, whereas MCA01 represents an average over approximately five months of continuous recordings. According to the ongoing analysis performed in the framework of the PROMUD project, the site appears to be affected by a switch in polarization direction (from N-S to about E-W), with a, roughly, daily periodicity. We are currently investigating the possible causes of this behavior, which may be endogenous or exogenous, natural or anthropogenic.

### 4.3. Transient Signals

Concerning the transient signals detected in the seismic background noise at the 2020 and 2023 vent areas, the western bubbling pool, and the northern emission zone, our analysis shows that they are characterized by high-frequency spectral content and distinct polarization features. Interestingly, the transients recorded in the 2020 and 2023 vent areas differ in both duration and polarization incidence angle: those in the 2020 vent field have short durations and nearly vertically polarization, whereas those near the 2023 mud cone last several seconds and are polarized in the horizontal plane. This could reflect differences in gas emission rates and source depths between the two vent systems. On the other hand, in the north area of the Maccalube, the horizontally polarized transients are likely linked to the bubbling at the surface of the western pool. The direct visual observation of active degassing and bubbling at the vents and pool during the seismic recordings supports their interpretation as indicators of local fluid-dynamical phenomena. In line with observations from other mud volcanic fields [18,25], these signals are likely associated with shallow, gas-driven processes and muddy fluid surface emissions. Notably, this work represents the first attempt to provide a detailed characterization of such transient signals at the Maccalube, in terms of both spectral attributes and polarization properties. This aspect is particularly relevant, as it allows not only to better constrain the background seismic fingerprint of the local dynamics, but also to establish direct comparisons with other, better-documented mud volcanoes, where similar signals have been reported [9,18,24,25,40,41,42]. In particular, analogies can be recognized in the duration, occurrence rate and the spectral features (Table 2), suggesting that these transient waveforms may represent a common expression of near-surface, gas-driven processes across different sedimentary volcanic areas.

Overall, although preliminary, the analyses conducted already provide meaningful insights into the structural and dynamic features of the Maccalube mud volcano system. A more detailed interpretation and the resolution of some open issues will nevertheless require further investigations, including integration with geological and geophysical datasets and an evaluation of the temporal stability of both HVSR curves and polarization parameters under varying environmental conditions. Finally, transient signals could be better characterized and explored through longer-term recordings and array-based analysis.

## 5. Conclusions and Future Perspectives

The DEMETRA experiment has provided a dense and spatially detailed dataset of ambient seismic noise in the Maccalube di Aragona, one of the most complex and active mud volcano systems in Italy. The high-resolution spatial coverage enabled the extraction of valuable information on both subsurface properties and ongoing fluid-related dynamics, demonstrating the effectiveness of dense noise-based surveys in such environments.

Mud volcanoes are increasingly recognized as key targets for multidisciplinary investigation due to their relevance to seismic hazard, environmental risk, and greenhouse gas emissions. In this context, the Maccalube area represents a site of particular interest, being a nature reserve where sedimentary volcanism poses potential threats to people and ecosystems. The integration of seismic monitoring into the broader framework of geohazard assessment in these environments is thus highly desirable.

Looking forward, further interpretation of the DEMETRA dataset will benefit from joint analyses with geological, geochemical, and hydrological data collected within the PROMUD project. In particular, time-lapse measurements and repeated noise surveys may help identify temporal variations and, potentially, precursory signatures of paroxysmal events. Moreover, advanced modeling of wave propagation and source processes will improve the characterization of transient signals and their relation to shallow fluid dynamics.

Two recent developments provide additional perspectives. First, a second multidisciplinary campaign, GEMINI (Geophysical, gEochemical and environMental Integrated iNvestigatIon), was carried out on 3–4 July 2025. This experiment, which involved seismologists, geochemists, physicists, and botanists, was specifically focused on the activity of the two main pools through a combination of seismic, geochemical, and bio-environmental measurements. Second, on 30 August 2025, a new overturning event occurred in the same sector affected by the 2014 paroxysm. Notably, the DEMETRA measurements had already revealed anomalous HVSR deamplifications in the vicinity of that area, suggesting that such features may deserve further investigation as possible markers of instability. The availability of multiparametric data recorded by the PROMUD instrumentation during this episode—an exceptional circumstance, since overturning events are rarely captured due to the lack of continuous monitoring at mud volcanoes—will allow a unique comparison of pre- and post-event conditions and is expected to provide key insights into the interpretation of such phenomena. A comprehensive multidisciplinary study on this event is currently in progress.

Overall, DEMETRA highlights the scientific and practical value of dense seismic noise monitoring in active mud volcano fields, providing a framework for future studies and long-term surveillance strategies in similar geologically active contexts.

## Figures and Tables

**Table 1:** Site names, location area, coordinates, date, and start/end local times of the recordings.

Site Name	Location Area	Latitude N (°)	Longitude E (°)	Elevation a.s.l. (m)	Day	Start	End
DMT01	2020 vents	37.37530	13.60001	300	22 April 2025	10:27:43	10:57:43
DMT02	2020 vents	37.37557	13.60036	292	22 April 2025	11:09:39	11:39:39
DMT03	Pools	37.37675	13.60008	294	22 April 2025	11:58:02	12:28:02
DMT04	Pools	37.37665	13.59960	303	22 April 2025	12:32:38	13:02:38
DMT05	2014 vents	37.37605	13.60006	305	22 April 2025	13:10:22	13:40:22
DMT06	2014 vents	37.37571	13.59970	294	22 April 2025	13:52:10	14:22:10
DMT07	2014 vents	37.37581	13.60018	297	22 April 2025	14:31:51	15:01:51
DMT08	2020 vents	37.37541	13.60043	303	22 April 2025	15:08:33	15:38:33
DMT09	2020 vents	37.37525	13.60031	305	22 April 2025	15:45:08	16:15:08
DMT10	2020 vents	37.37501	13.60036	306	22 April 2025	16:24:42	16:54:42
DMT11	MCA08	37.37464	13.60024	293	22 April 2025	17:04:26	17:34:26
DMT12	2023 vents	37.37427	13.60211	288	23 April 2025	09:59:15	10:29:15
DMT13	2023 vents	37.37420	13.60177	298	23 April 2025	10:37:57	11:07:57
DMT14	2023 vents	37.37420	13.60126	292	23 April 2025	11:14:25	11:44:25
DMT15	2023 vents	37.37403	13.60196	289	23 April 2025	11:55:01	12:25:01
DMT16	South emission	37.37327	13.60157	290	23 April 2025	12:36:30	13:06:30
DMT17	MCA10	37.37519	13.60161	293	23 April 2025	13:18:49	13:48:49
DMT18	North emission	37.37727	13.60162	295	23 April 2025	14:04:42	14:34:42
DMT19	MCA01	37.38026	13.59406	275	23 April 2025	14:52:48	15:22:48
DMT20	2020 vents	37.37504	13.60006	304	23 April 2025	15:40:08	16:10:08
DMT21	2020 vents	37.37493	13.60057	307	23 April 2025	16:13:39	16:43:39

**Table 2:** Comparison of the main characteristics of the transient signals recorded at Maccalube di Aragona and across different mud volcanic fields. The year reported in the Date column refers to the time of observation. When available, the Duration/Description column reports the nomenclature or classification of the signals adopted by the authors.

Mud Volcano	Date	Duration/Description	Inter-Event Times	Spectral Content	Polarization Incidence Angle
Maccalube di Aragona 2020 vents	2025	<1 s	20–40 s	20–40 Hz	20–30°
Maccalube di Aragona 2023 vents	2025	50–80 s		5–20 Hz	80°
Maccalube di Aragona pools	2025	1–2 s	Irregular	5–20 Hz	80°
Maccalube di Aragona North emission	2025	1–2 s	100 s	40–60 Hz	60–80°
Maccalube di Santa Barbara [9]	2017	10 s	5 min	5–10 Hz + overtones	
Salse di Nirano [41]	2012	20 s	60 s	10–25 Hz	
Salse di Nirano [41]	2013	20 s	100 s	10–25 Hz	
Salse di Nirano [18]	2016	50–60 s Drumbeats 1	3 min	10–45 Hz	
Salse di Nirano [18]	2016	4–5 s Drumbeats 2	22 min	5–45 Hz	
Salse di Nirano [18]	2019	3–4 s Drumbeats 3	1 min	10–150 Hz	
Salse di Nirano [24,25]	2021 2023	5–30 s Drumbeats Single high-energy events (0.2–0.3 s) randomly occur within the sequence	Irregular	10–30 Hz	70°
Salse di Nirano [24,25]	2021 2023	10–50 s Drumroll Sequences of identical short pulses (<1 s) regularly distributed in time	0.3 s for pulses in the drumroll sequence	5–30 Hz	70°
Dashgil [40]	2006	1.5 s Class A	18.5 s	3 5 Hz	60°
Dashgil [40]	2006	1.5 s Class B	14.5 s	3 5 Hz	30°
Dashgil [40]	2007	1.5 s Class B sub-a	5 min	4.5 Hz	10–20°
Dashgil [40]	2007	1.5 s Class B sub-b	4 min	5.5 Hz	20–60°
Dashgil [40]	2007	1.5 s Class C sub-c	7 min	6.5 Hz	60°
Håkon Mosby, Barents Sea [42]	2009	<1 s		3–20 Hz	
Håkon Mosby, Barents Sea [42]	2009	6.7 h Tremor swarms	13.7 h	4–5, 8–10 11–13 Hz	

## Data Availability

Data used in this study are acquired in the framework of the 2023–2025 PROMUD project and are available from the corresponding author on reasonable request.

## Appendix A

Recommendation for the interpretation of the HVSR results according to SESAME guidelines [29].

**Reliable Curve Criteria**
  *f*_0_ > 10/l_w_
  n_c_ (*f*_0_) > 200
  σ_A_(*f*) < 2 for 0.5 *f*_0_ < *f* < 2 f_0_ if *f*_0_ > 0.5 Hz or σ_A_(*f*) < 3 for 0.5 *f*_0_ < *f* < 2 *f*_0_ if *f*_0_ < 0.5 Hz

**Clear Peak Criteria (CPC)—at least 5 out of 6 criteria to be fulfilled**
  (1) ∃ *f*^−^ ∈ [*f*_0_/4, *f*_0_] | A_H/V_(*f*^−^) < A_0_/2
  (2) ∃ *f*^+^ ∈ [*f*_0_, 4 *f*_0_] | A_H/V_(*f*^+^) < A_0_/2
  (3) A_0_ > 2
  (4) *f*_peak_[A_H/V_(*f*) ± σ_A_(*f*)] = *f*_0_ ± 5%
  (5) σ_f_ < ε(*f*_0_)
  (6) σ_A_(*f*_0_) < θ (*f*_0_)

l_w_ = window length
  n_w_ = number of windows selected for the average H/V curve
  n_c_ = l_w_ × n_w_ × *f*_0_ = number of significant cycles
  *f* = current frequency
  *f*_sensor_ = sensor cut-off frequency
  *f*_0_ = H/V peak frequency
  σ_f_ = standard deviation of H/V peak frequency (*f*_0_ ± σ_f_)
  ε (*f*_0_) = threshold value for the stability condition σ_f_ < ε(*f*_0_)
  A_0_ = H/V peak amplitude at frequency *f*_0_
  A_H/V_ (*f*) = H/V curve amplitude at frequency *f*
  *f*^−^ = frequency between *f*_0_/4 and *f*_0_ for which A_H/V_(*f*^−^) < A_0_/2
  *f*^+^ = frequency between *f*_0_ and 4 *f*_0_ for which A_H/V_(*f*^+^) < A_0_/2
  σ_A_ (*f*) = “standard deviation” of A_H/V_ (*f*), σ_A_ (*f*) is the factor by which the mean A_H/V_(*f*) curve should be multiplied or divided
  σ_logH/V_ (*f*) = standard deviation of the logA_H/V_(*f*) curve, σ_logH/V_ (*f*) is an absolute value which should be added to or subtracted from the mean logA_H/V_(*f*) curve
  θ (*f*_0_) = threshold value for the stability condition σ_A_(*f*) < θ(*f*_0_)

**Table A1:** HVSR analysis results: site name, percentage of used waveforms, resonance frequency and its standard deviation, peak amplitude, and SESAME [29] Clear Peak Criteria (1–6) compliance.

Site	Data %	*f*_0_ ± σ (Hz)	A_0_	CPC 1	CPC 2	CPC 3	CPC 4	CPC 5	CPC 6
DMT01	97	0.41 ± 0.02	2.39	YES	YES	YES	NO	YES	YES
DMT02	98	0.41 ± 0.24	2.39	YES	YES	YES	NO	NO	YES
DMT03	95	0.78 ± 0.34	2.17	NO	NO	YES	NO	NO	YES
DMT04	95	4.06 ± 2.26	3.06	NO	NO	YES	NO	NO	YES
DMT05	95	10.0 ± 2.3	3.16	NO	NO	YES	NO	NO	YES
DMT06	98	4.31 ± 2.3	3.99	YES	NO	YES	NO	NO	YES
DMT07	97	3.75 ± 2.24	3.32	YES	NO	YES	NO	NO	YES
DMT08	97	4.63 ± 3.23	2.70	YES	NO	YES	NO	NO	YES
DMT09	97	4.91 ± 1.24	2.77	YES	NO	YES	NO	NO	YES
DMT10	97	5.31 ± 1.06	3.88	YES	NO	YES	NO	NO	YES
DMT11	97	6.13 ± 2.38	2.79	NO	NO	YES	NO	NO	YES
DMT12	97	0.47 ± 6.23	1.64	NO	NO	NO	NO	NO	YES
DMT13	92	0.31 ± 0.08	1.82	YES	YES	NO	NO	NO	YES
DMT14	92	0.47 ± 0.03	2.42	NO	YES	YES	NO	YES	YES
DMT15	93	5.56 ± 1.13	1.75	NO	NO	NO	NO	NO	YES
DMT16	92	0.47 ± 4.67	1.56	NO	NO	NO	NO	NO	YES
DMT17	98	2.47 ± 0.38	3.29	YES	NO	YES	NO	NO	YES
DMT18	98	1.97 ± 0.10	4.07	YES	NO	YES	NO	YES	YES
DMT19	92	1.19 ± 0.27	5.76	YES	NO	YES	NO	NO	YES
DMT20	93	11.97 ± 5.81	3.91	NO	NO	YES	NO	NO	YES
DMT21	95	10.88 ± 2.03	3.07	NO	NO	YES	NO	NO	YES
